# Plasma Biomarkers Screening by Multiplex ELISA Assay in Patients with Advanced Non-Small Cell Lung Cancer Treated with Immune Checkpoint Inhibitors

**DOI:** 10.3390/cancers13010097

**Published:** 2020-12-31

**Authors:** Adrien Costantini, Paul Takam Kamga, Catherine Julie, Alexandre Corjon, Coraline Dumenil, Jennifer Dumoulin, Julia Ouaknine, Violaine Giraud, Thierry Chinet, Martin Rottman, Jean-François Emile, Etienne Giroux Leprieur

**Affiliations:** 1 Department of Respiratory Diseases and Thoracic Oncology, APHP—Hôpital Ambroise Pare, 92100 Boulogne-Billancourt, France; adrien.costantini@aphp.fr (A.C.); coraline.dumenil@aphp.fr (C.D.); jennifer.dumoulin@aphp.fr (J.D.); julia.ouaknine@aphp.fr (J.O.); violaine.giraud@aphp.fr (V.G.); thierry.chinet@aphp.fr (T.C.); 2 EA 4340 BECCOH, UVSQ, Université Paris-Saclay, 92100 Boulogne-Billancourt, France; takam.paul@gmail.com (P.T.K.); catherine.julie@aphp.fr (C.J.); jean-francois.emile@uvsq.fr (J.-F.E.); 3 Department of Pathology, APHP—Hôpital Ambroise Pare, 92100 Boulogne-Billancourt, France; alexandre.corjon@aphp.fr; 4 Department of Microbiology, APHP—Hôpital Raymond Poincaré, 92380 Garches, France; martin.rottman@aphp.fr; 5 UMR 1173, UVSQ, Université Paris-Saclay, 78180 Montigny-le-Bretonneux, France

**Keywords:** non-small cell lung cancer, immune checkpoint inhibitor, biomarker, plasma, resistance, toxicity, hepatocyte growth factor, Fibroblast growth factor, interleukine-12

## Abstract

**Simple Summary:**

There is an unmet need for new predictive biomarkers associated with efficacy and immune-related toxicity of immune checkpoint inhibitors (ICIs) in non-small cell lung cancer (NSCLC). In this study, we performed multiplex ELISA screening in plasma from 35 consecutive patients with advanced NSCLC treated with nivolumab or pembrolizumab, allowing large-scale screening for 48 cytokines involved in immune response and tumour proliferation. We found an association between ICIs efficacy and three cytokines: soluble hepatocyte growth factor (sHGF), soluble Fibroblast Growth Factor (sFGF) and interleukine-12 (IL-12). Moreover, TNF-α, IL-16, IL-12p40 and MCP3 were candidate biomarkers for predicting grade 3–4 immune-related toxicity. This exploratory study shows the potential role of new plasma biomarkers in advanced NSCLC treated with ICIs.

**Abstract:**

Immune checkpoint inhibitors (ICIs) are commonly used in patients with advanced non-small cell lung cancer (NSCLC). An unmet need remains for new biomarkers associated with ICIs. In this study, consecutive patients with advanced NSCLC treated with nivolumab or pembrolizumab were included. Plasma at ICIs initiation was prospectively collected and a multiplex ELISA assay testing 48 cytokines and growth factors was performed. Exploratory endpoints were the association between plasma biomarkers with outcome and grade III–IV immune related adverse events (irAEs). Thirty-five patients were included. Patients without clinical benefit (*n* = 22) had higher pre-ICI soluble Hepatocyte Growth Factor (sHGF) (210.9 vs. 155.8 pg/mL, *p* = 0.010), lower pre-ICI soluble Fibroblast Growth Factor (sFGF) (4.0 vs. 4.8 pg/mL, *p* = 0.043) and lower pre-ICI interleukine-12 (IL-12) (1.3 vs. 2.2 pg/mL, *p* = 0.043) concentrations. Patients with early progression (*n* = 23) had higher pre-ICIs sHGF (206.2 vs. 155.8 pg/mL, *p* = 0.025) concentrations. Patients with low sHGF levels at ICIs initiation had longer progression-free survival and overall survival than those with high sHGF levels: respectively 2.5 vs. 8.0 months (*p* = 0.002), and 5.5 vs. 35.0 months (*p* = 0.001). TNF-α, IL-16, IL-12p40 and MCP3 were associated with high grade irAEs. This study shows the potential association between several plasma biomarkers with outcome and grade 3–4 IrAEs in advanced NSCLC treated with ICIs.

## 1. Introduction

Lung cancer is the leading cause of cancer related death worldwide [1]. At the advanced stage, its prognosis is bleak with limited efficacy of cytotoxic chemotherapy (CT). Immune checkpoint inhibitors (ICIs), humanised monoclonal antibodies targeting notably programmed death 1 (PD-1) or programmed death ligand 1 (PD-L1), have recently been developed. PD-L1 and programmed death-ligand 2 (PD-L2) are membranous proteins expressed by malignant cells that interact with PD-1 expressed by T-cells. When PD-L1/PD-L2 and PD-1 bind, the T-cells’ cytotoxic anti-tumour activity is down-regulated. By blocking the interaction between PD-L1 and PD-1, ICIs restore cytotoxic immune response. ICIs have shown their efficacy in advanced non-small cell lung cancer (NSCLC). Nivolumab, an anti-PD-1 antibody, is currently used for second-line treatment in Anaplastic Lymphoma Kinase (*ALK*) and Epidermal Growth Factor Receptor (*EGFR)* wild-type advanced NSCLC [2,3]. Pembrolizumab, (anti-PD-1 antibody) is used for first-line treatment in *ALK* and *EGFR* wild-type advanced NSCLC that have a high (≥50%) PD-L1 expression on tumour cells as determined by immunohistochemistry (IHC) [4]. Pembrolizumab can also be used in the first-line setting in association with platinum-pemetrexed doublet CT independent of PD-L1 expression determined by IHC [5,6]. Other drugs such as atezolizumab or durvalumab (anti-PD-L1 monoclonal antibodies) have also shown their efficacy in different settings [7,8,9]. However, the use of PD-L1 expression as a predictive biomarker remains challenging, as some patients experience tumour response with low/negative PD-L1 expression [2,3,7,9]. Furthermore, PD-L1 expression as determined by IHC can vary within one tumour sample, between two different locations of the same tumour [10,11,12,13,14,15,16] as well as over time, notably after CT [17,18]. There is currently an unmet need for biomarkers to better select patients who will benefit from ICIs. Other than tissue-based biomarkers, plasma-based biomarkers are being investigated [19,20] as plasma has the advantage of being easily accessible, allows sequential analysis during follow-up and reflects the different tumour clones present throughout the body.

In this study, we aimed to perform baseline plasma biomarker screening using a multiplex ELISA assay in patients with advanced NSCLC receiving anti-PD-1 monoclonal antibodies nivolumab or pembrolizumab. The aim was to perform exploratory analyses of the potential association between baseline biomarker levels and clinical outcomes and toxicity. Due to the exploratory nature of the study, no primary endpoint was chosen.

## 2. Results

### 2.1. Patients

Between March 2014 and February 2018, 86 patients received nivolumab or pembrolizumab for treatment of NSCLC. Thirty-five patients (41%) signed the consent form and had plasma available for analyses. Their characteristics are presented in Table 1. Gender was evenly distributed among the patients (51% male), they were mainly current or former smokers (83%) and with adenocarcinoma histology (77%). All patients were negative for Epidermal Growth Factor Receptor (EGFR) sensitising mutations and Anaplastic Lymphoma Kinase (ALK) translocation. ICIs were given as first-line treatment (pembrolizumab, *n* = 8) because of high PD-L1 NSCLC, in the second-line setting (nivolumab, *n* = 21) or beyond (nivolumab, *n* = 6). At the time of cut-off, median follow-up was 47.0 months (IQR 36.0–70.0), twenty-four patients (69%) were deceased due to tumour progression, seven (20%) had controlled disease under ICIs and four (11%) were receiving further treatment after ICI failure. Objective Response Rate (ORR) under immunotherapy was 49% with 17 patients presenting with partial response under ICIs as best response, 5 (14%) presenting with stable disease and 13 (37%) presenting with progressive disease as best tumour response. Median Progression Free Survival (PFS) under ICIs was 4.0 months (IQR 2.0–7.0) and median Overall Survival (OS) was 21.0 months (IQR 10.0–35.0). Twenty-two patients (63%) did not present with clinical benefit whilst receiving immunotherapy, twelve (34%) presented with clinical benefit and one patient (3%) could not be evaluated. Twenty-three patients (66%) presented with early progression and twelve patients (34%) presented with late progression while receiving ICIs.

### 2.2. Plasma Biomarkers

**Figure 1:**
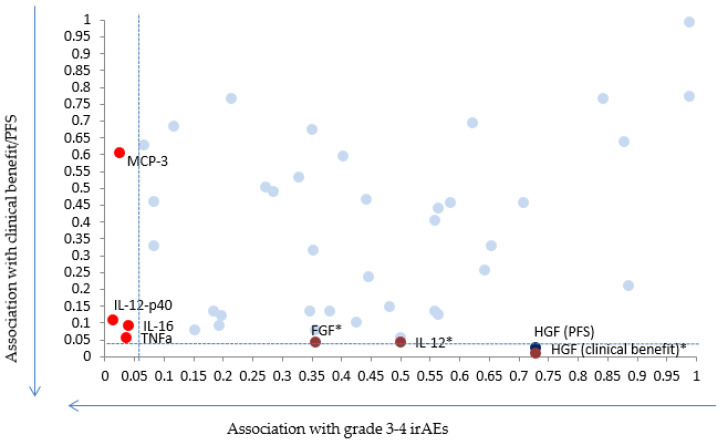
Scatter plot showing *p*-values for the 48 chemokines and cytokines with regards to grade 3–4 immune adverse events (irAEs) (*x* axis) and Progression Free Survival (PFS) or clinical benefit (*y* axis). * Indicates biomarkers for which results remain significant after applying correction for multiple testing.

Amongst the tested cytokines, three seemed to be associated with outcome in our cohort: soluble HGF (sHGF), soluble FGF (sFGF) and IL-12 (Figure 1). Table S1 shows the raw data for the measured biomarkers in pg/mL.

**Figure 2:**
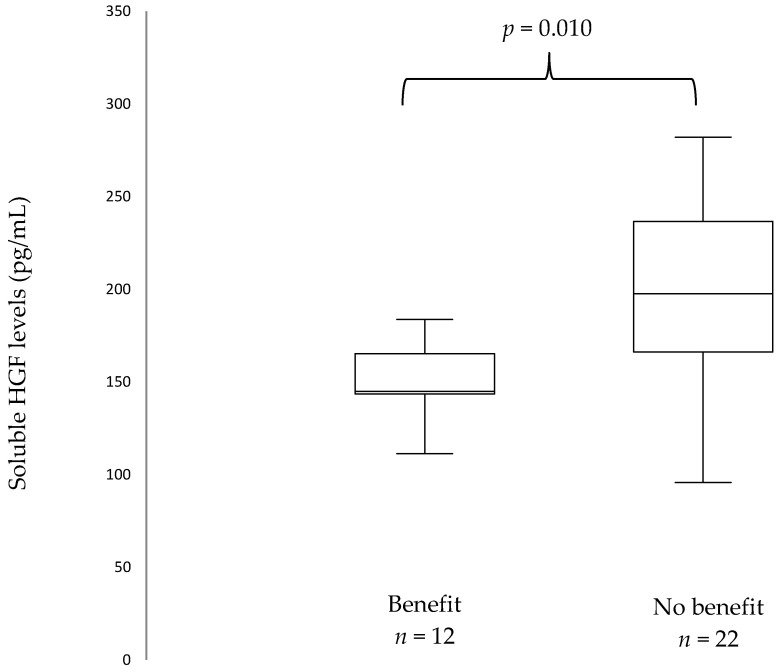
Box plots showing baseline soluble Hepatocyte Growth Factor (sHGF) levels in patients with and without clinical benefit. The median is indicated by the line within the box, the boundaries of the box indicate the 25th and 75th percentile and the whiskers the 10th and 90th percentile.

At ICI initiation, median sHGF was 171.35 pg/mL (IQR 144.05–210.5). A stepwise approach was used: first, all biomarkers were tested with regards to progression pattern (early vs. late progression). Patients who presented with early progression (*n* = 23) had higher sHGF levels at ICIs initiation than patients with late progression (*n* = 12): 206.2 pg/mL vs. 155.8 pg/mL, *p* = 0.025 (Figure S1). When Benjamini–Hochberg correction for multiple testing was applied, this did not translate into significant results. Secondly, only biomarkers with promising results with regards to progression pattern were selected. Patients who did not present with clinical benefit (*n* = 22) had higher sHGF levels at immunotherapy initiation than patients who presented with clinical benefit (*n* = 12): 210.9 pg/mL versus (vs.) 155.8 pg/mL, *p* = 0.010 (Figure 2). When Benjamini–Hochberg correction for multiple testing was applied using a false discovery rate (Q) of 5%, results remained significant.

This did not translate into ORR with no statistically significant difference in sHGF levels in patients presenting with stable or progressive disease (*n* = 18) as compared to those presenting with response (*n* = 17): 207.1 pg/mL vs. 137.3 pg/mL, *p* = 0.145.

**Figure 3:**
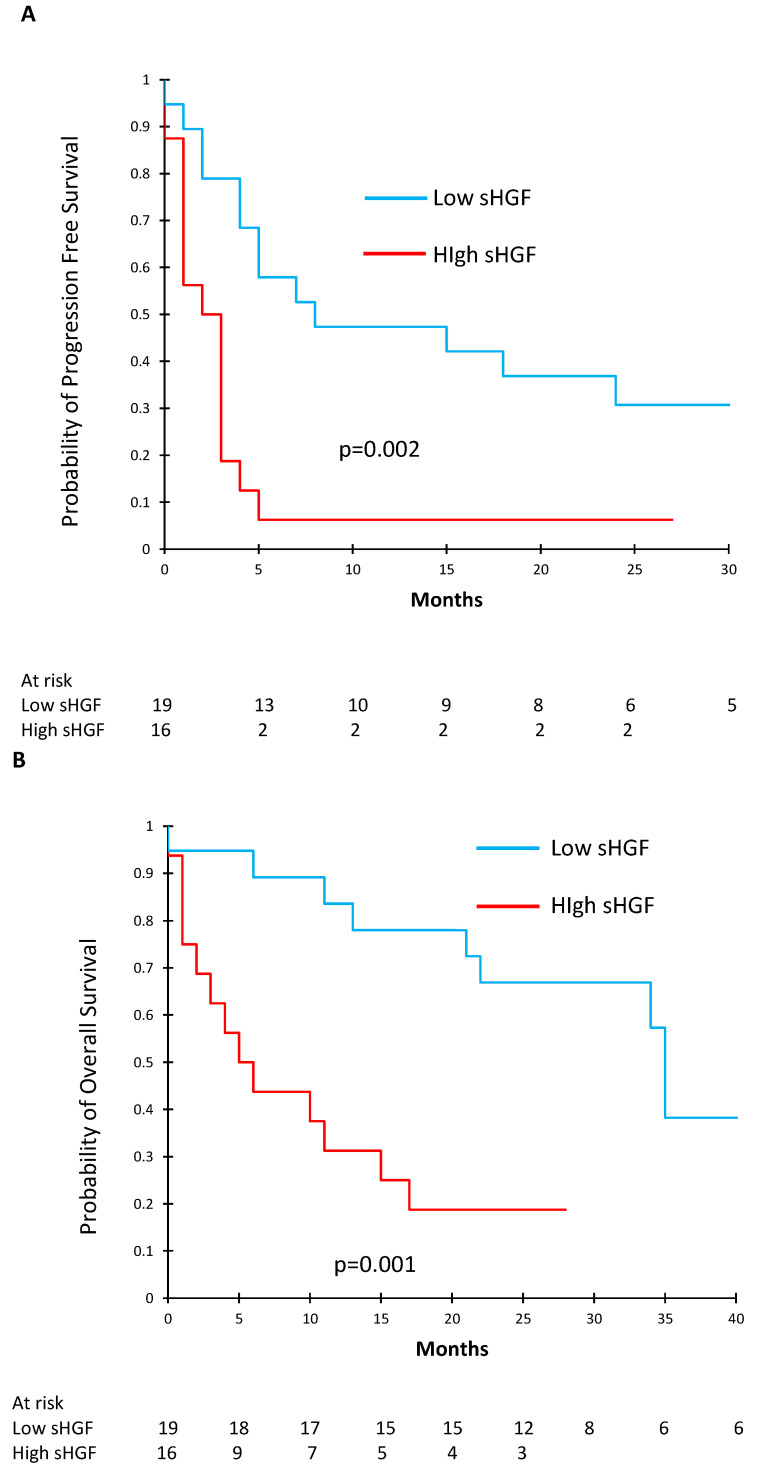
Kaplan–Meier curves showing progression free survival (PFS) (**A**) and overall survival (**B**) according to baseline sHGF levels.

To further determine the effect of sHGF on survival, we used ROC curves to determine the optimal sHGF cut-off in order to separate our patients into two groups: high and low baseline sHGF concentrations. Table 2 shows the characteristics of the patients with high (*n* = 16) and low (*n* = 19) pre-ICI sHGF levels. Of note, patients with low pre-ICI sHGF levels had better PS than patients with high sHGF levels (PS 0–1.89% vs. 59%, *p* = 0.049). We found that an sHGF level of 171.35 pg/mL offered a sensitivity of 70% and a specificity of 83% to determine PFS. The area under the curve (AUC) was 0.73 (Figure S2). Using this cut-off, we constructed Kaplan–Meier survival curves and compared PFS and OS between patients with high (*n* = 16) and low (*n* = 19) baseline sHGF levels. PFS was significantly shorter in the high baseline sHGF group: median PFS under immunotherapy was 2.5 months [95% CI (1.0–3.0)] vs. 8.0 months [95% CI (4.0—not reached (NR))], *p* = 0.002 (Figure 3A). In the same way, OS was significantly shorter in the high sHGF group: median OS under immunotherapy was 5.5 months [95% CI (2.0–15.0)] vs. 35.0 months [95% CI (22.0—NR)], *p* = 0.001 (Figure 3B).

### 2.3. Fibroblast Growth Factor (FGF)

Patients who did not present with clinical benefit had lower sFGF levels at immunotherapy initiation than patients who presented with clinical benefit: 4.0 pg/mL vs. 4.8 pg/mL (*p* = 0.043) and this result remained significant after applying correction for multiple testing. This did not translate into significant results with regards to ORR (4.2 pg/mL for non-responders vs. 4.4 pg/mL for responders, *p* = 0.380) or progression pattern (4.0 pg/mL for early progression vs. 4.8 pg/mL for late progression, *p* = 0.056).

### 2.4. Interleukine-12 (IL-12)

Patients who did not present with clinical benefit had lower IL-12 levels at immunotherapy initiation than patients who presented with clinical benefit: 1.3 pg/mL vs. 2.2 pg/mL (*p* = 0.043) and this result remained significant after applying correction for multiple testing. This did not translate into significant results with regards to ORR (1.9 pg/mL for non-responders vs. 1.9 pg/mL for responders, *p* = 0.572) or progression pattern (1.4 pg/mL for early progression vs. 2.2 pg/mL for late progression, *p* = 0.080).

### 2.5. Immune-Related Adverse Events (irAEs)

Six patients (17%) presented with grade III–IV toxicity whilst receiving immunotherapy: auto-immune kidney failure/nephritis (*n* = 1), auto-immune cholangitis (*n* = 1), interstitial pneumonia/pneumonitis (*n* = 2), one patient with multiple toxicities (encephalitis, skin, arthro-myalgia), arthro-myalgia (*n* = 1). Of the tested biomarkers, higher levels of TNF-α (*p* = 0.036), IL-16 (*p* = 0.040), IL-12p40 (*p* = 0.015) and MCP3 (*p* = 0.025) were significantly associated with grade 3–4 irAEs under immunotherapy. When correction for multiple testing was applied, results did not remain significant.

## 3. Discussion

Although ICIs have transformed patient care across numerous cancer types, their efficacy remains sub-optimal and the need for biomarkers in order to better select patients who will benefit from them is essential. In this pilot study, we performed large baseline plasma biomarker screening using a multiplex ELISA assay in patients with advanced NSCLC treated with nivolumab or pembrolizumab. We were able to show a significant association between clinical benefit under ICIs and levels of sHGF, sFGF and IL-12. Results with regards to the progression pattern were promising but significance was lost when correction for multiple testing was applied. Similarly, we found a potential association high immune toxicities and levels of IL-16, TNF-α, IL-12p40 and MCP3.

HGF is a polypeptide growth factor that belongs to the plasminogen family. It is a disulfide-linked α–β heterodimer consisting of a 69 kDa α-chain and a 34 kDa β-chain. HGF is produced by mesenchymal cells (stromal cells and fibroblasts) in an inactive single-chain precursor of 728 amino acids (pro-HGF), which is then activated by posttranslational conversion by a serine protease in areas of tissue injury. The *MET* gene encodes for c-MET, a high-affinity receptor of HGF [21,22] that is, in a physiological setting, expressed on epithelial cells. HGF specifically activates c-MET receptor tyrosine kinase activity. The HGF/c-MET pathway can induce epithelial-to-mesenchymal transition (EMT), motility, proliferation and is involved in regeneration and tissue repair [23,24].

In cancer, c-MET activation promotes communication between mesenchymal cells and epithelial cells, tissue infiltration, cancer cell proliferation, and the induction of angiogenesis [25].

This is the first study to report results regarding pre-ICI sHGF levels and outcomes in patients with advanced NSCLC treated with ICIs. We found that patients who presented with clinical benefit and late progression had lower pre-ICI sHGF levels than patients who did not. Furthermore, patients with low pre-ICI sHGF levels as determined by ROC curves presented with significantly longer PFS and OS than patients with high pre-ICI sHGF levels.

This result is in line with what has been shown in previous studies investigating sHGF. In resectable NSCLC, sHGF levels were associated with survival [26,27]. In patients with advanced NSCLC receiving cytotoxic CT, sHGF levels were evaluated at different time-points during follow-up (pre-treatment, response-evaluation 1–2 months after treatment initiation, best tumour response and disease progression) in 55 patients [28]. Positive-sHGF at response-evaluation predicted poor PFS compared with negative-sHGF in first-line (median, 153.5 vs. 288.0; *p* < 0.05) and second-line treatment (87.0 vs. 219.5; *p* = 0.01). Multiple Cox proportional hazards models showed significant independent associations between poor PFS and positive-sHGF at response-evaluation (hazard ratio, 4.24; 95% CI, 2.05 to 9.46; *p* < 0.01) and positive-sHGF at pre-treatment or at response-evaluation predicted poor PFS (35.0 vs. 132.0; *p* < 0.01, 50.0 vs. 215.0; *p* < 0.01, respectively).

Soluble HGF has also been extensively investigated in patients with EGFR mutant advanced NSCLC treated with EGFR tyrosine kinase inhibitors (TKIs). Preclinical and clinical studies have shown that sHGF is associated with poor outcome with EGFR TKIs, notably through the activation of the MET pathway [29,30,31,32]. Some recent reports have evaluated sHGF levels in patients receiving ICIs. Kubo et al. [33] published a retrospective study on 29 patients with metastatic melanoma treated with pembrolizumab or nivolumab. Patients without tumour response had higher baseline sHGF levels than patients with tumour response (*p* = 0.00124). Furthermore, patients with low sHGF levels showed longer OS (*p* = 0.039; HR 0.3125, 95% CI 0.1036–0.9427) and PFS (*p* = 0.0068; HR 0.2087, 95% CI 0.06525–0.6676) than those with high sHGF levels.

The interaction between the HGF/c-MET pathway and anti-tumour immune-response is complex and yet to be fully understood. It has been shown [34,35] that PD-L1 expression occurs more frequently with MET activation. In fact, PD-L1 expression was positively associated with MET gene amplification in 389 NSCLC samples and, in a separate study of 155 resected NSCLC tumour samples. There is evidence that the HGF/c-MET axis interacts with immuno-modulation: HGF treated monocytes have immuno-suppressive phenotypes, HGF promotes immuno-tolerant CD4+ response, C-MET+ CD 8 + T cells produce more inflammatory cytokines and HGF treated antigen presenting cells attenuate cytokine production and memory T cell formation. Also, it is established that HGF induces macrophage transition to the M2 phenotype, which is pro-generative. However, several studies have suggested that HGF could also have a pro-immune role [36,37] and further research is needed to better understand the exact role of HGF in anti-tumour immune response.

Amongst the other tested biomarkers, statistically significant results were found with sFGF and IL-12 with regards to clinical benefit. Patients with higher baseline sFGF levels presented with clinical benefit as compared to those with lower baseline sFGF levels. Although FGF signalling is associated with cellular proliferation, survival, migration and differentiation, there is also evidence that the FGF pathway can act in a tumour suppressive manner in some circumstances [38].

Finally, we found that patients who did not present with clinical benefit had lower IL-12 levels at immunotherapy initiation than patients who presented with clinical benefit. This finding can be explained by the fact that IL-12 has a clear anti-tumour activity through the activation of T and natural killer (NK) lymphocytes leading to the production of interferon gamma (IFNγ) [39].

The onset of irAEs is difficult to predict, and although grade 3–4 adverse events remain relatively infrequent, they can be severe, impacting quality of life, sometimes life-threatening, and can lead to treatment interruption. Predicting the onset of the irAEs is a major challenge and the use of soluble biomarkers has already been partly explored in a previous study [20] finding that low sPD-L2, low IL-2 and high IFN-g levels in patients with advanced NSCLC treated with nivolumab were associated with grade 3–4 toxicities. In this study, we found several candidate biomarkers to predict irAEs. Higher levels of TNF-α (*p* = 0.036), IL-16 (*p* = 0.040), IL-12p40 (*p* = 0.015) and MCP3 (*p* = 0.025) were all seemingly associated with grade 3–4 irAEs under immunotherapy. If confirmed, these results could help predict which patients are at risk for high-grade toxicity as early as the beginning of treatment, leading to closer follow-up of such patients.

Our work has several limitations. This was a small exploratory monocentric pilot study, with a possibility of lack of power for some statistical analyses. However, when correction for multiple testing was applied some relevant results remained significant. Finally, it is difficult to differentiate the prognostic and the predictive role of these biomarkers, as no validation cohort with a control group was used and further studies are needed to confirm these preliminary results.

## 4. Materials and Methods

### 4.1. Experimental Design

This study was an exploratory study, based on the analysis of consecutive patients in the Department of Respiratory Medicine and Thoracic Oncology (APHP—Ambroise Paré Hospital) treated by nivolumab or pembrolizumab for stage III (non-irradiable) or IV NSCLC between 2014 and 2018, and for whom plasma samples at diagnosis were available. Exploratory endpoints were ORR, PFS, OS, clinical benefit, early or late progression and grade 3–4 toxicity, according to plasmatic concentrations of a panel of potential biomarkers.

### 4.2. Patients and Plasma

Tumour response was evaluated every eight (nivolumab) or nine (pembrolizumab) weeks using iRECIST criteria [40]. Medical records were reviewed and data retrospectively extracted on clinical and pathological features as well as treatment history. Plasma samples were drawn before initiating immunotherapy (C1). Briefly, two 10 mL-EDTA tubes of peripheral blood were taken, plasma was isolated after centrifugation within one hour and immediately stored at −80 °C.

### 4.3. Ethical Considerations

All included patients signed an informed consent allowing blood to be drawn and stored within the Centre de Ressources Biologiques (CRB) of the Ambroise Paré University Hospital during their follow-up and treatment. The protocol was approved by the Institutional Review Board CPP IDF n_8 (ID CRB 2014-A00187-40).

### 4.4. Outcomes

ORR was defined as the proportion of patients who presented with partial or complete response whilst receiving ICIs. Clinical benefit was defined as complete response, partial response or stability, according to iRECIST [40], lasting 6 months or more after initiation of immunotherapy. PFS was defined as the time between ICI initiation and tumour progression or death. OS was defined as the time between ICI initiation and death. Patients were defined as presenting with early progression as opposed to late progression if disease progression occurred within six months of initiating ICIs.

Immune related adverse events (irAEs) were assessed using Common Terminology Criteria for Adverse Events (CTCAE v4.0).

### 4.5. Multiplex ELISA Technique

Multiplex ELISA was performed on the patients’ plasma samples using a commercial kit and according to the manufacturer’s guidelines (Bio-Plex-Pro^TM^ Human Cytokines Assay, Bio-Rad). This assay allows testing of 48 chemokines, cytokines and growth factors in plasma samples. The assay principle is that of a sandwich ELISA: capture antibodies directed against the desired biomarker are covalently coupled to fluorescently dyed magnetic microspheres (beads) each with a distinct colour code or spectral address. After several wash series, the final detection complex is formed with the addition of streptavidin-phycoerythrin (SA-PE) conjugate with phycoerythrin serving as a fluorescent indicator. Data are drawn using an automated reader, a red and green laser illuminates the fluorescent dyes within each bead allowing to provide bead classification and PE excitation, which is detected by a photomultiplier tube (PMT). Data are presented as median fluorescence intensity (MFI) as well as concentration in pg/mL. The concentration of analyte bound to each beach is proportional to the MFI of the reporter signal. All samples, standards and negative controls were tested in duplicate. For all the 48 tested cytokines, the related mean intra-assay CV (coefficient of variation) ranged from 1.7 to 5.0%, and inter-assay CV from 1.2 to 7.9%.

The following proteins were tested in this assay: Interleukin-1a (IL-1a), IL-1b, IL-1ra,IL-2ra,IL-2,IL-3, IL-4,IL-5,IL-6,IL-7, IL-8,IL-9,IL-10,IL-12, IL-12p40,IL-13,IL-15,IL-17, IL-16,IL-18, Eotaxin, Fibroblast Growth Factor (FGF), Granulocyte-Colony Stimulating Factor (G-CSF), Granulocyte Macrophage–Colony Stimulating Factor (GM-CSF), Interferon gamma (IFN-g), IP-10, Monocyte chemoattractant protein 1 (MCP-1), MCP-3,Macrophage Inflammatory Protein 1 alpha (MIP-1a),MIP-1b, Platelet Derived Growth Factor-bb (PDGF-bb), Regulated upon Activation, Normal T Cell Expressed and Presumably Secreted (RANTES), Tumour Necrosis Factor–alpha (TNF-a), Vascular Endothelial Growth Factor (VEGF), Cutaneous T-cell-Attracting Chemokine (CTACK), Growth Regulated Oncogene-alpha (GRO-a), Hepatocyte Growth Factor (HGF), Interferon-alpha 2 (IFN-a2), Leukemia inhibitory factor (LIF), Monocyte-Colony Stimulating Factor (M-CSF), Macrophage migration Inhibitory Factor (MIF), Monokine Induced by Gamma interferon (MIG), beta-Nerve Growth Factor (b-NGF), Stem Cell Factor (SCF), Stem Cell Growth Factor-beta (SCGF-b), Stromal Cell-Derived Factor-1 (SDF-1a), Tumour Necrosis Factor-beta (TNF-b), TNF-related apoptosis-inducing ligand (TRAIL).

### 4.6. Statistical Analysis

Median soluble concentrations of all the tested biomarkers were analysed according to ORR, PFS, OS, clinical benefit, early or late progression response profile and grade 3–4 toxicity.

The comparison of median biomarker levels between groups was performed using the Mann–Whitney test and interquartile range (IQR) is given for each value.

A stepwise approach was adopted. First, all biomarkers were tested with regards to PFS. Biomarkers who presented with significant or closely significant *p*-values were then tested in a second step with regards to clinical benefit. In both cases, correction for multiple testing was applied using the Benjamini–Hochberg [41] method correction for multiple testing and setting a false discovery rate (Q) at 5%.

The Receiving Operating Curve (ROC) method was used to determine a cut-off level for each biomarker with a significant difference for endpoints with the Mann–Whitney test. The Kaplan–Meier method was used to determine OS and PFS. Comparison between survival curves was performed using a log-rank method. Data analysis was computed using XLStat v 19.4 (Addinsoft). *p*-values were considered significant if <0.05.

## 5. Conclusions

This is the first study to show the prognostic value of sHGF in patients with advanced NSCLC treated with ICIs. This finding is in line with previous data reported in localised NSCLC, EGFR mutated NSCLC and advanced NSCLC treated with CT. Furthermore, this study brings original data with regards to the use of FGF or IL-12 as potential biomarkers of ICI efficacy, and IL-16, TNF-α, IL-12p40 and MCP3 as biomarkers of irAEs.

These preliminary results need to be validated in a large prospective population, including patients treated with ICI-CT combo treatment.

## Figures and Tables

**Table 1:** Patients’ characteristics.

Characteristics	All Patients (*n* = 35)
Sex	
Male *n* (%)	18 (51%)
Female *n* (%)	17 (49%)
Age at diagnosis, median (range)	67 (37–84)
Smoking history	
Non-smoker, *n* (%)	6 (17%)
Smoker, *n* (%)	29 (83%)
Histology	
Non squamous	27 (77%)
Squamous	5 (14%)
Other	3 (9%)
Stage at diagnosis	
I-II, *n* (%)	2 (6%)
III-IV, *n* (%)	33 (94%)
PS at ICIs initiation	
0–1, *n* (%)	26 (74%)
2, *n* (%)	9 (26%)
Type of ICIs	
Pembrolizumab (1st line)	8 (26%)
Median number of infusions (range)	9 (2–37)
Nivolumab (≥2nd line)	27 (77%)
Median number of infusions	8 (1–76)

PS: performance status, ICIs: immune checkpoint inhibitors.

**Table 2:** Characteristics of patients with high and low pre-ICI sHGF levels.

Characteristics	Patients with Low sHGF (*n* = 18)	Patients with High sHGF (*n* = 17)	*p*-Value
Sex			*p* = 0.238
Male *n* (%)	11 (61%)	7 (41%)	
Female *n* (%)	7 (39%)	10 (59%)	
Age at diagnosis, median (range)	66.5 (48–84)	70 (37–82)	*p* = 0.457
Smoking history			*p* = 0.061
Non-smoker, *n* (%)	1 (6%)	5 (29%)	
Smoker, *n* (%)	17 (94%)	12 (71%)	
Histology			*p* = 0.657
Non squamous	15 (83%)	12 (70%)	
Squamous	2 (11%)	3 (18%)	
Other	1 (6%)	2 (12%)	
Stage at diagnosis			*p* = 0.157
I-II, *n* (%)	2 (11%)	0 (0%)	
III-IV, *n* (%)	16 (89%)	17 (100%)	
PS at ICI initiation			*p* = 0.042
0–1, *n* (%)	16 (89%)	10 (59%)	
≥2, *n* (%)	2 (11%)	7 (41%)	
Type of ICI			*p* = 0.110
Pembrolizumab (1st line)	4 (22%)	4 (24%)	
Nivolumab (≥2nd line)	11 (61%)	10 (59%)	

PS: performance status, ICIs: immune checkpoint inhibitors.

## Data Availability

Data are available upon reasonable request.

## Supplementary Materials

The following are available online at https://www.mdpi.com/2072-6694/13/1/97/s1, Figure S1: Box plots showing baseline soluble HGF levels in patients with early and late progression. The median is indicated by the line within the box, the boundaries of the box indicate the 25th and 75th percentile and the whiskers the 10th and 90th percentile, Figure S2: ROC curve used to determine the sHGF cut-off level, Table S1: Raw values of the different measured biomarkers.
