# Supplementary material for: Plasma Biomarkers Screening by Multiplex ELISA Assay in Patients with Advanced Non-Small Cell Lung Cancer Treated with Immune Checkpoint Inhibitors
Source: Cancers (Basel). 2020 Dec 31;13(1):97. doi: 10.3390/cancers13010097 (PMC7795942; doi:10.3390/cancers13010097)
Supplement: Supplementary file 1 [file cancers-13-00097-s001.pdf]

Supplementary Materials: Plasma Biomarkers Screening by Multiplex ELISA Assay in Patients with Advanced Non-small Cell Lung Cancer Treated with Immune Checkpoint Inhibitors

Adrien Costantini, Paul Takam Kamga, Catherine Julie, Alexandre Corjon, Coraline Dumenil, Jennifer Dumoulin, Julia Ouaknine, Violaine Giraud, Thierry Chinet, Martin Rottman, Jean-François Emile and Etienne Giroux Leprieur

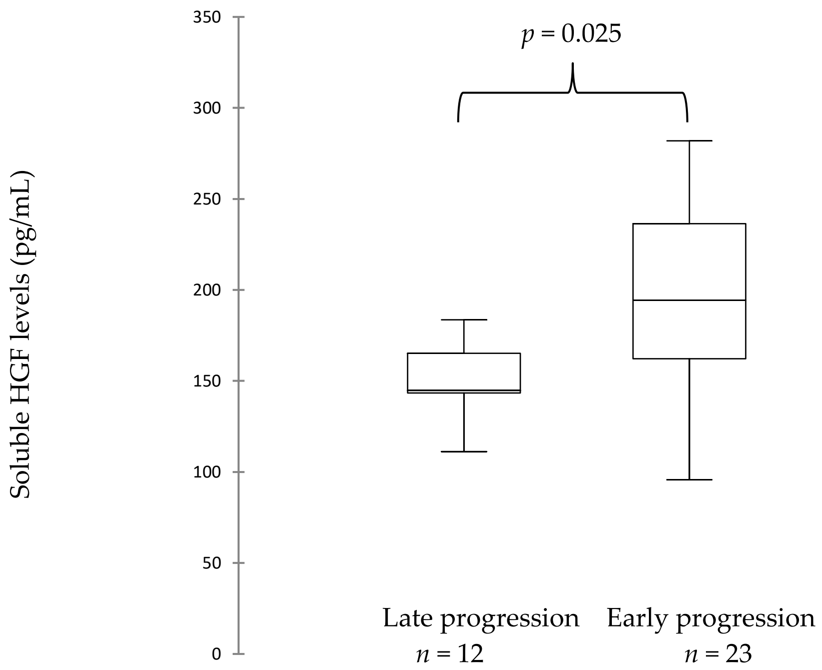

Figure S1. Box plots showing baseline soluble HGF levels in patients with early and late progression. The median is indicated by the line within the box, the boundaries of the box indicate the 25th and 75th percentile and the whiskers the 10th and 90th percentile.

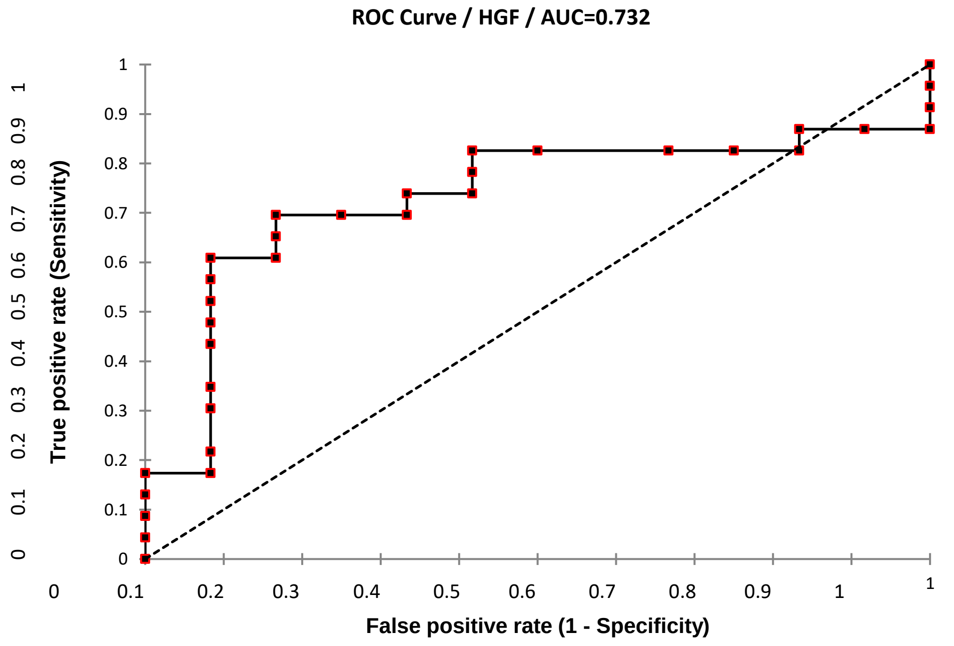

Figure S2. ROC curve used to determine the sHGF cut-off level.

Table S1. Raw values of the different measured biomarkers.

IL-1a	IL-1b	IL-1Ra	IL-2Ra	IL-2	IL-3	IL-4	IL-5
46.37	16.85	94.26	32.12	2.4	1.82	0.75	1.73
4.47	2.49	32.43	28.42	0.53	0.16	1.04	OOR <
2.9	13.78	136.53	25.4	0.24	0.1	0.4	OOR <
5.33	1.55	60.76	19.76	1.13	0.23	0.75	OOR <
8.32	1.24	29.61	37.21	1.79	0.42	0.82	1.09
2.66	1.85	17.09	23.18	0.09	0.09	OOR <	OOR <
2.66	0.64	11.53	17.3	OOR <	0.05	OOR <	OOR <
4.35	0.76	28.16	20.2	0.35	0.15	0.55	OOR <
2.9	0.96	397.01	68.08	OOR <	0.06	OOR <	OOR <
9.08	8.31	70.83	43.48	1.65	0.25	0.58	0.91
6.07	1.32	43.32	39.15	0.74	0.16	0.15	OOR <
6.32	3.9	48.56	53.45	2.13	0.3	0.94	OOR <
3.87	2.73	28.16	41.77	0.74	0.14	0.05	OOR <
9.58	3.25	53.61	34.61	2.27	0.36	0.63	OOR <
5.33	2.93	46.12	53.39	1.09	0.18	0.02	OOR <
4.6	1.65	66.81	23.57	0.78	0.14	0.18	OOR <
3.62	2.01	40.46	27.07	OOR <	0.12	0.34	OOR <
2.9	3.09	29.97	41.6	0.74	0.09	0.32	OOR <
4.35	5.82	33.13	19.43	0.88	0.1	0.32	OOR <
3.14	3.05	40.78	42.23	0.74	0.14	0.42	OOR <
4.6	6.83	49.77	74.78	1.02	0.18	0.4	OOR <
4.84	3.49	63.53	36.59	0.6	0.13	0.15	OOR <
3.14	3.98	241.57	41.77	OOR <	0.05	0.08	OOR <
5.58	1.69	34.5	29.42	0.99	0.2	0.78	OOR <
4.11	4.14	38.51	26.74	0.31	0.13	0.45	OOR <
6.82	1.41	50.36	43.6	1.09	0.25	0.55	OOR <
4.35	4.42	25.94	24.51	0.53	0.13	0.32	OOR <
5.33	9.22	39.16	48.37	0.95	0.19	0.42	OOR <

19.81	11.41	45.5	53.62	3.62	1.09	0.44	12.02
6.32	3.05	115.16	22.68	1.09	0.25	0.75	1.26
3.87	7.63	24.42	24.29	0.6	0.13	0.23	OOR <
5.09	1.69	33.82	30.94	1.37	0.25	1.36	0.01
2.66	0.6	17.52	17.14	OOR <	0.07	0.34	OOR <
5.58	2.69	52.44	30.43	1.09	0.27	0.82	OOR <
4.35	3.05	134.19	46.24	0.38	0.14	0.29	OOR <
**IL-6**	**IL-7**	**IL-8**	**IL-9**	**IL-10**	**IL-12**	**Il-12p40**	**IL-13**
3.47	19.82	9.22	94.69	1.61	9.93	93.27	5.51
0.2	30.68	4.01	70.16	OOR <	0.99	1.06	0.93
0.75	18.58	13.39	59.29	OOR <	0.76	OOR <	2.64
0.93	22.03	14.65	69.87	1.05	1.3	4.03	1.35
2.17	27.82	5.13	62.83	0.48	2.9	5.24	0.78
0.18	8.83	2.39	57.23	OOR <	0.54	OOR <	0.97
0.01	12.51	0.49	54.12	OOR <	0.54	OOR <	0.31
0.16	24.7	1.59	54.56	OOR <	0.84	OOR <	0.57
0.14	10.95	5.23	55.45	OOR <	0.39	OOR <	0.59
0.39	15.57	4.35	62.09	0.2	1.5	5.85	4.83
0.54	11.47	5.62	54.41	OOR <	1.18	2.83	0.89
0.34	22.76	6.79	76.29	0.68	2.26	15.15	2.93
0.24	10.95	2.68	50.41	0.09	0.99	2.83	1.25
0.5	21.05	5.91	59.59	OOR <	2.66	10.16	1.8
0.45	8.83	6.69	60.33	OOR <	1.15	4.93	1.59
1.08	16.08	4.35	48.92	OOR <	1.15	2.83	1.05
0.08	18.33	2.34	54.56	OOR <	0.69	OOR <	1.73
0.93	16.08	2.09	59.88	OOR <	0.76	OOR <	1.72
0.22	14.56	3.22	54.56	OOR <	0.76	OOR <	1.89
0.62	18.08	6.98	57.97	0.09	1.15	OOR <	5.1
0.08	15.57	3.13	56.64	0.2	0.91	OOR <	1.79
0.12	11.99	3.96	56.04	OOR <	0.76	OOR <	1.73
0.7	10.42	18.54	48.32	OOR <	0.54	OOR <	2.22
0.35	23.73	2.39	59.59	0.26	1.15	OOR <	2.57
0.29	18.58	3.27	56.93	OOR <	0.99	OOR <	1.97
1.82	25.91	7.96	60.18	2.13	1.46	1.06	0.79
0.33	18.08	1.99	61.36	OOR <	0.84	OOR <	1.35
0.58	17.58	10.58	63.42	OOR <	1.3	0.49	1.63
4.53	20.06	2.98	87.18	0.91	10.11	9.54	4.63
0.43	24.94	3.08	67.53	0.39	2.74	1.64	1.93
0.29	12.51	2.29	62.98	OOR <	0.84	1.06	4
0.29	28.78	4.84	72.06	1.26	1.3	6.46	2.08
0.08	15.32	1.09	55.9	OOR <	0.46	OOR <	0.55
0.45	32.09	3.96	58.41	0.58	1.78	OOR <	1.07
0.05	13.79	5.96	53.08	OOR <	0.54	0.49	2.64
**IL-15**	**IL-16**	**IL-17**	**IL-18**	**Eotaxin**	**FGF**	**G-CSF**	**GM-CSF**
16.69	15.21	4.33	13.37	3.79	11.08	528.41	1.95
OOR <	15.42	4.58	6.51	18.5	3.6	OOR <	0.32
OOR <	6.8	2.55	11.11	7.55	3.76	OOR <	0.55
OOR <	16.1	3.43	14.67	8	4.22	OOR <	0.71
OOR <	10.44	3.83	4.5	7.7	4.67	OOR <	1.03
OOR <	7.03	0.77	8.41	3.81	3.28	OOR <	0.29

OOR <	11.22	1.69	4.48	4.67	2.58	OOR <	0.07
OOR <	12.07	3.93	6.89	9.18	3.92	OOR <	0.28
OOR <	6.33	1.05	6.58	3.15	3.19	OOR <	0.1
OOR <	44.78	3.04	17.7	2.68	5.65	OOR <	1.69
OOR <	9.46	1.79	7.81	1.42	4.52	OOR <	0.55
OOR <	15.9	3.14	4.27	2.68	4.22	OOR <	1.52
OOR <	18.51	1.14	8.56	1.53	3.28	OOR <	0.55
OOR <	14.25	3.33	7.87	1.5	6.05	OOR <	0.99
OOR <	16.44	1.23	11.87	0.91	4.3	OOR <	0.55
OOR <	9.93	1.05	11.21	0.91	4.07	OOR <	0.37
OOR <	23.96	2.55	5.67	5.89	3.28	OOR <	0.5
OOR <	10.2	1.88	11.93	4.48	3.76	OOR <	0.42
OOR <	12.65	2.17	8.97	5.11	3.6	OOR <	0.31
OOR <	7.81	1.23	3.83	5.23	3.6	OOR <	1.68
OOR <	25.84	1.98	12.52	7.13	3.84	OOR <	0.9
OOR <	7.3	1.98	10.42	3.89	4.07	OOR <	0.47
OOR <	4.53	1.51	5.14	3.5	1.82	OOR <	0.29
OOR <	14.8	4.43	9.14	9.7	5.1	OOR <	0.81
OOR <	11.32	2.94	9.3	8.16	3.28	OOR <	0.57
OOR <	13.16	3.73	11.37	6.38	5.24	OOR <	0.5
OOR <	9.39	3.33	3.79	6.19	4.37	OOR <	0.47
OOR <	11.46	2.7	14.82	5.46	4.07	OOR <	1.11
19.76	15.96	4.23	13.8	6.06	8.53	OOR <	3.94
OOR <	16.72	4.43	7.36	10.57	5.92	OOR <	1.15
OOR <	10.61	1.88	5.68	4.41	2.94	OOR <	0.83
OOR <	16	4.88	6.13	14.93	3.92	OOR <	1.22
OOR <	10.1	2.46	3.92	7.77	2.4	OOR <	0.1
OOR <	18.85	4.33	7.36	9.69	4.37	OOR <	0.57
OOR <	15.76	3.14	39.02	6.44	3.92	OOR <	0.56

**IFN-g**	**IP-10**	**MCP-1**	**MCP-3**	**MIP-1a**	**MIP-1b**	**PDGF-bb**	**RANTEs**
9.33	74.54	66.57	14.42	1.52	22.24	68.27	247.57
6.78	113	5.48	1.04	0.49	23.23	30.7	1289.63
4.72	37.67	12.08	1.04	0.41	21.11	24.62	1184.01
5.16	71.93	14.46	2.06	0.72	21.95	99.35	1407.43
7.51	60.63	3.05	2.88	0.45	21.57	164.24	757.61
1.71	117.99	3.58	0.67	0.22	19.98	106.59	1320.19
0.85	58.69	2.76	0.39	0.21	19.77	103.88	731.28
2.32	64.59	7.84	1.16	0.32	18.43	214.57	906.91
12.95	117.18	2.58	0.67	0.33	21.53	212.29	1065.01
7.95	38.62	4.27	2.21	0.57	21.89	99.35	1616.42
5.16	OOR <	6.66	1.6	0.39	18.57	88.6	1052.88
9.84	58.83	8.49	2.41	0.61	20.82	30.18	956.19
4.72	7.37	6.91	1.27	0.38	18.03	4.82	957.4
7.14	38.31	4.38	3.15	0.73	19.35	38.92	656.67
7.66	22.96	3.67	1.91	0.69	20.69	14.72	433.52
3.97	12.34	16.34	1.81	0.51	19.61	25.16	1299
5.53	173.52	4.47	2.21	0.55	11.56	91.82	492.25
4.27	62.41	4.17	0.86	0.35	19.47	65.18	1129.11
2.77	88.01	3.73	1.1	0.25	19.92	82.82	781.7
7.22	32.79	1.86	1.21	0.38	19.61	122.23	867.03

10.13	211.57	10.72	1.76	0.89	20.21	26.49	766.06
5.53	118.22	4.11	1.6	0.43	18.62	102.07	888
8.38	75.84	8.66	12.29	0.54	18.34	26.49	518.05
4.72	211.07	7.64	1.7	0.35	19.55	50.41	850.95
3.22	133.1	6.07	1.16	0.41	19.14	49.17	906.6
6.92	201.04	5.93	2.01	0.66	23.25	453.53	965.44
3.67	61.32	3.09	1.16	0.38	20.81	120.01	773.68
7.51	352.93	9.81	1.81	0.58	21.13	15.28	1093.15
13.46	381.23	6.85	5.27	0.54	23.59	123.11	1218.76
5.16	34.44	4.56	1.91	0.67	20.14	170.86	941.8
3.82	102.86	3.35	0.92	0.3	20.92	98.44	1256.2
6.56	147.94	7.89	1.81	0.51	22.56	267.8	1482.71
3.07	107.96	5.06	0.24	0.32	21.14	28.87	844.15
6.26	96.36	5.45	2.11	0.61	17.9	142	896.73
6.48	73.5	3.54	1.16	0.46	18.6	32	568.18
**TNF-a**	**TNF-b**	**VEGF**	**CTACK**	**GRO-a**	**HGF**	**IFN-a2**	**LIF**
41.73	13.94	OOR <	300.06	62.8	447.23	15.45	47.12
17.23	6.62	OOR <	293.95	OOR <	172.76	2.16	4.52
14.07	OOR <	OOR <	168.71	13.05	207.19	1.56	0.77
22.55	OOR <	OOR <	240.51	31.98	236.49	2.63	5.47
20.79	OOR <	OOR <	118.15	47.04	102.74	2.82	9.27
12.71	OOR <	OOR <	139.22	OOR <	112.59	0.91	1.08
9.48	OOR <	OOR <	204.86	OOR <	95.72	0.49	OOR <
13.62	OOR <	OOR <	183.56	OOR <	145.46	1.67	2.01
10.41	OOR <	OOR <	127.43	OOR <	371.6	1.03	0.46
28.24	OOR <	OOR <	222.95	77.25	144.05	2.91	8.95
20.57	OOR <	OOR <	102.8	19.12	131.83	1.67	3.58
27.8	1.56	OOR <	326.28	43.97	143.11	3.62	9.59
19.01	OOR <	OOR <	277.26	OOR <	111.18	1.56	2.64
30.83	0.01	OOR <	184.81	31.58	161.93	3.45	9.9
25.62	OOR <	OOR <	154.23	18.52	166.64	2.06	3.89
18.34	OOR <	OOR <	196.09	11.39	200.59	1.97	2.95
19.46	OOR <	OOR <	202.89	OOR <	159.58	1.56	3.11
17.67	OOR <	OOR <	358.99	OOR <	144.05	1.72	2.48
13.62	OOR <	OOR <	147.44	6.02	171.35	1.67	2.48
15.43	OOR <	OOR <	81.58	OOR <	188.79	1.87	3.26
25.62	OOR <	OOR <	419.82	OOR <	194.45	2.45	4.21
14.07	OOR <	OOR <	151.73	31.58	213.8	1.56	1.39
13.16	OOR <	OOR <	85.32	OOR <	281.92	1.03	0.77
17.67	OOR <	OOR <	216.68	12.24	164.76	2.54	3.89
15.88	OOR <	OOR <	92.28	10.49	164.28	1.67	2.32
22.55	OOR <	OOR <	142.08	54.95	262.98	2.45	5.47
15.88	OOR <	OOR <	288.75	OOR <	207.19	1.87	1.7
19.9	OOR <	OOR <	232.08	50.54	248.78	2.54	4.68
40.67	10.37	OOR <	178.19	103.67	183.6	3.09	33.91
21.89	OOR <	OOR <	200.38	118.75	143.58	2.59	15.95
15.88	OOR <	OOR <	192.33	28.2	185.49	1.56	2.32
20.34	OOR <	OOR <	377.87	43.97	160.05	2.82	7.05
9.02	OOR <	OOR <	183.03	84.81	101.81	0.68	0.77
20.79	OOR <	OOR <	207.19	OOR <	247.84	2.63	4.52

12.25	OOR <	OOR <	311.37	OOR <	236.49	1.97	3.26

**M-CSF**	**MIF**	**MIG**	**b-NGF**	**SCF**	**SCGF-b**	**SDF-1a**	**TRAIL**
15.84	442.44	661.93	OOR <	19.53	17581.03	80.73	58.28
7.88	215.82	258	OOR <	15.55	14154.67	216.58	18.83
9.34	135.51	62.08	OOR <	16.43	17178.98	139.1	20.11
9.87	280.36	67.16	OOR <	13.97	11895.73	158.2	24.23
10.26	167.28	85.76	OOR <	8.54	6977.99	138.44	23.52
5.44	307.51	294.83	OOR <	6.9	6883.81	121.95	27.32
6.3	174.27	117.02	OOR <	10.17	7221.06	114.81	35.85
5.66	212.04	188.09	OOR <	9.91	9236.03	112.97	18.83
8.82	305.81	117.47	OOR <	6.42	10852.69	135.48	18.01
13.02	520.04	282.35	OOR <	38.74	10359.17	58.44	46.98
7.77	493.67	131.51	OOR <	13.07	8049.57	37.04	24.7
3.24	403.65	222.75	OOR <	26.91	18119.94	70.95	16.51
8.62	276.57	317.31	OOR <	23.69	5106.29	35.66	30.66
9.62	190.02	780.86	OOR <	16.43	12286.97	47	30.42
12.48	324.09	294.46	OOR <	21.9	6498.76	43.98	17.2
6.71	378.5	216.74	OOR <	10.01	8467.41	33.88	15.47
12.93	253.02	308.46	OOR <	30.2	13807.81	125.43	49.7
13.24	240.51	114.35	OOR <	22.52	4653.36	148.59	21.63
7.94	269.66	274.04	OOR <	13.33	11845.34	186.25	28.99
8.71	214.81	80.48	OOR <	13.62	9894.55	96.32	22.58
11.66	289.07	1454.37	OOR <	26.62	13781.18	188.51	19.76
11.15	182.56	164.9	OOR <	14.94	11046.92	106.28	32.59
4.33	178.86	68.92	OOR <	10.04	8967.78	102.41	21.16
11.58	226.72	390.95	OOR <	22.39	9464.69	204.81	38.16
8.57	239.3	200.45	OOR <	11.39	7612.33	134.5	29.71
13.27	215	155.56	OOR <	12.23	13658.15	132.36	16.27
8.16	217.21	102.12	OOR <	21.67	6877.84	154.79	28.03
10.79	202.26	195.48	OOR <	10.24	14838.9	137.29	27.55
20.77	141.32	561.11	OOR <	16.13	9941.97	198.52	34.04
9.59	368.44	50.01	OOR <	13.45	13352.97	116.81	14.2
5.77	311.78	536.23	OOR <	11.33	8095.49	135.16	26.84
5.39	516.86	321.98	OOR <	20.21	8555.38	220.29	26.36
3.96	248.49	250.65	OOR <	11.14	7450.3	134	22.58
11.49	245.83	160.14	OOR <	17.07	17875.29	100.22	21.63
22.62	181.52	166.35	OOR <	11.88	18048.75	112.97	23.05

ORR<: below the detection threshold.

Copyright: © 2020 by the authors. Licensee MDPI, Basel, Switzerland. This article is an open access article distributed under the terms and conditions of the Creative Commons Attribution (CC BY) license (http://creativecommons.org/licenses/by/4.0/).
